# Global Analysis of Posttranscriptional Gene Expression in Response to Sodium Arsenite

**DOI:** 10.1289/ehp.1408626

**Published:** 2014-11-21

**Authors:** Lian-Qun Qiu, Sarah Abey, Shawn Harris, Ruchir Shah, Kevin E. Gerrish, Perry J. Blackshear

**Affiliations:** 1 Laboratory of Signal Transduction, National Institute of Environmental Health Sciences (NIEHS), National Institutes of Health (NIH), Department of Health and Human Services (DHHS), Research Triangle Park, North Carolina, USA; 2 Social and Scientific Systems Inc., Research Triangle Park, North Carolina, USA; 3 Molecular Genomics Core Laboratory, NIEHS, NIH, DHHS, Research Triangle Park, North Carolina USA; 4 Department of Medicine, and; 5 Department of Biochemistry, Duke University Medical Center, Durham, North Carolina, USA

## Abstract

**Background:**

Inorganic arsenic species are potent environmental toxins and causes of numerous health problems. Most studies have assumed that arsenic-induced changes in mRNA levels result from effects on gene transcription.

**Objectives:**

We evaluated the prevalence of changes in mRNA stability in response to sodium arsenite in human fibroblasts.

**Methods:**

We used microarray analyses to determine changes in steady-state mRNA levels and mRNA decay rates following 24-hr exposure to noncytotoxic concentrations of sodium arsenite, and we confirmed some of these changes using real-time reverse-transcription polymerase chain reaction (RT-PCR).

**Results:**

In arsenite-exposed cells, 186 probe set–identified transcripts were significantly increased and 167 were significantly decreased. When decay rates were analyzed after actinomycin D treatment, only 4,992 (9.1%) of probe set–identified transcripts decayed by > 25% after 4 hr. Of these, 70 were among the 353 whose steady-state levels were altered by arsenite, and of these, only 4 exhibited significantly different decay rates between arsenite and control treatment. Real-time RT-PCR confirmed a major, significant arsenite-induced stabilization of the mRNA encoding δ aminolevulinate synthase 1 (*ALAS1*), the rate-limiting enzyme in heme biosynthesis. This change presumably accounted for at least part of the 2.7-fold increase in steady-state *ALAS1* mRNA levels seen after arsenite treatment. This could reflect decreases in cellular heme caused by the massive induction by arsenite of heme oxygenase mRNA (*HMOX1*; 68-fold increase), the rate-limiting enzyme in heme catabolism.

**Conclusions:**

We conclude that arsenite modification of mRNA stability is relatively uncommon, but in some instances can result in significant changes in gene expression.

**Citation:**

Qiu LQ, Abey S, Harris S, Shah R, Gerrish KE, Blackshear PJ. 2015. Global analysis of posttranscriptional gene expression in response to sodium arsenite. Environ Health Perspect 123:324–330; http://dx.doi.org/10.1289/ehp.1408626

## Introduction

Inorganic arsenic compounds are potent environmental toxicants. Humans are exposed to various forms of arsenic mainly through oral consumption of contaminated water, food, or drugs as well as via inhalation of arsenic-containing dust or smoke in agricultural and industrial settings (Jomova et al. 2011; Nordstrom 2002). Chronic exposure to arsenic has been associated with many adverse health effects in humans, including cancers, diabetes mellitus, and diseases of the cardiovascular, nervous, and reproductive systems (Centeno et al. 2002; Jomova et al. 2011). Millions of people worldwide are exposed to elevated arsenic concentrations in their drinking water due to naturally high levels in groundwater, placing them at risk for developing arsenic-related cancers and other diseases (Jomova et al. 2011; Nordstrom 2002).

The precise mechanisms of disease development following arsenic exposure are not completely understood. The toxicity of arsenic has been associated with the activation or inhibition of various biochemical events, including signal transduction and cell proliferation and differentiation (Druwe and Vaillancourt 2010; Hughes et al. 2011). Most studies have assumed that arsenic-induced changes in gene expression result from transcriptional activation or repression following arsenic exposure. Arsenic has been shown to alter the DNA binding of transcription factors known to regulate many inducible genes, including *SP1*, *JUN*, and nuclear factor-kappa B (*NFKB*) (Hamilton et al. 1998). Genome-wide microarray analyses have identified arsenic-enriched transcription networks for proteins involved in common pathophysiological processes such as tumorigenesis, inflammation, cell cycle regulation, immune function, and diabetes (Ahlborn et al. 2008; Andrew et al. 2008; Benton et al. 2011). These effects have been attributed to changes of transcription rates, interference with DNA repair mechanisms, activation of cellular differentiation, and histone modification (Gadhia et al. 2012).

Posttranscriptional regulation is another important locus of gene expression control, but the influence of arsenic on posttranscriptional regulation of gene expression has remained largely unexplored. This is despite the well-known effect of arsenic compounds to stimulate the formation of stress granules and P (processing) bodies, both thought to be involved in localizing mRNA decay (Buchan 2014). Posttranscriptional regulation involves the steps of mRNA splicing, processing, nuclear export, translation, sequestration, and degradation. The importance of these steps in gene regulation has been discussed widely; for example, in a recent review, Carpenter et al. (2014) highlighted the mechanisms and consequences of posttranscriptional regulation in the innate immune response. Many of these processes and events are regulated by RNA binding proteins. For example, arsenic was shown to increase the stability of *STAT1* (signal transducer and activator of transcription 1) mRNA, apparently mediated through the RNA binding protein nucleolin (Zhang et al. 2006). More recently, a study in HepG2 cells demonstrated that the inhibitory effect of arsenic on catalase expression was regulated at both transcriptional and posttranscriptional levels (Kim et al. 2011).

The purpose of our study was to perform a genome-wide analysis of sodium arsenite–induced changes in gene expression in human diploid fibroblasts and to determine whether these changes could be due, at least in part, to changes in mRNA stability. We chose diploid human foreskin fibroblasts as the test cells because skin is sensitive to the effects of chronic arsenic exposure and it is where the first manifestations of exposure often appear (Huang et al. 2004; Rossman et al. 2004). We also used diploid, nontransformed cells in an attempt to mimic normal human stromal cells as opposed to cancer cells. We used a noncytotoxic dose of 1 μM sodium arsenite (Burnichon et al. 2003; Rea et al. 2003) to avoid secondary effects of cytotoxicity on gene expression. Arsenite and other soluble salts are major environmental contaminants in groundwater worldwide and are useful for biochemical studies because of water solubility and rapid transport into cells (Druwe and Vaillancourt 2010).

## Materials and Methods

*Cell culture and stimulation*. BJ human diploid foreskin fibroblasts (catalog no. CRL-2522) were obtained from ATCC. Cells were maintained in Eagle’s minimal essential medium supplemented with 10% fetal bovine serum, 100 U/mL penicillin, and 100 μg/mL streptomycin. Cells were plated into 100-mm dishes until they reached 80% confluence, and then various concentrations of sodium arsenite (catalog no. 71287; Sigma Chemical Co.) or vehicle (water) were added to the medium for 24 hr. We determined cytotoxicity of various concentrations of sodium arsenite after 24 hr using propidium iodide staining followed by flow cytometric analysis (Ross et al. 1989).

*RNA extraction*. After 24 hr of arsenite or vehicle treatment, cells were washed twice with phosphate-buffered saline, and total cellular RNA was isolated using the Illustra RNAspin MiniRNA Isolation Kit (GE Healthcare) according to the manufacturer’s instructions. Residual genomic DNA was removed by on-column digestion with RNase-free DNase I supplied with the kit. Quality and integrity of RNA samples was checked on denaturing formaldehyde/agarose gels stained with acridine orange. After fibroblasts were treated with sodium arsenite or water (vehicle) for 24 hr, actinomycin D was added to the culture medium at a final concentration of 5 μg/mL; treated cells were then harvested at 0, 1, 2, 3, and 4 hr for RNA extraction. This concentration of actinomycin D has been validated in other cell types to be both effective and noncytotoxic for these relatively short times (Lai et al. 2006; Qiu et al. 2012).

*Microarray analysis*. Microarray analysis of gene expression was conducted using GeneChip Human Genome U133A Plus 2.0 GeneChip® Arrays (Affymetrix). This entire human genome array contains > 54,000 probe sets, reflecting > 38,500 genes and approximately 47,000 transcripts (Affymetrix 2003). For our analyses, 1 μg of total RNA was reverse transcribed to synthesize first-strand cDNA, which was then converted into a double-stranded cDNA template for *in vitro* transcription and labeling, following the Affymetrix One-Cycle cDNA Synthesis protocol. Pooled amplified biotin-cRNA (12.5 μg) was then fragmented, and 10 μg was hybridized onto each GeneChip 3´ expression array for 16 hr at 45°C in a rotating hybridization oven, using the Affymetrix Eukaryotic Target Hybridization controls and protocol. Array slides were stained with streptavidin/phycoerythrin using a double-antibody staining procedure; slides were then washed using the EukGE-WS2v5 protocol of the Affymetrix Fluidics Station FS450 for antibody amplification. Arrays were scanned in an Affymetrix Scanner 3000, and data were obtained using GeneChip Command Console software (AGCC, version 1.1). Data processing, normalization, and error modeling were performed using the Rosetta Resolver system (version 7.2; https://www.rosettacommons.org/software). Pathway and functional analyses of the differentially expressed transcripts were performed using Ingenuity Pathway Analysis (IPA; version 5.5; Ingenuity Systems®) and Partek Genomics Suite (Partek Inc.).

Although using microarrays for the analysis of mRNA levels has various disadvantages, as reviewed recently (Bradford et al. 2010; Malone and Oliver 2011), we have used it previously, in conjunction with actinomycin D treatment, as an initial screen that allowed us to discover a number of transcripts that were stabilized in the absence of the mRNA-binding protein tristetraprolin (TTP) (Lai et al. 2006). These transcripts were then validated by more quantitative techniques (i.e., northern blotting). We used a similar experimental paradigm in the present studies.

*Microarray data accession number*. The microarray analysis results have been deposited in the National Center for Biotechnology Information (NCBI) Gene Expression Omnibus (GEO) Profiles database (accession no. 57051).

*Real-time reverse transcription polymerase chain reaction (RT-PCR)*. Selected transcripts were analyzed by real-time RT–PCR. For each sample, 1 μg of total cellular RNA was reverse transcribed using oligo(dT)_12–18_ primers and SuperScript III Reverse Transcriptase (Invitrogen) following the manufacturer’s protocol. All cDNAs were diluted and subjected to real-time PCR using SYBR Green master mix and the ABI Prism 7900 Sequence Detection System (both from Applied Biosystems). Transcript-specific primers were designed and validated for their amplification efficiency prior to being used in the study, and are listed in Supplemental Material, Table S1. Relative transcript abundance was determined by normalizing values to the β-actin (*ACTB*) transcript as an internal control, which was then used to calculate the fold changes relative to vehicle control, or the relative abundance of each transcript compared with the pre-actinomycin D values, according to the ΔΔCt method (Pfaffl 2001).

*Statistical analysis*. Each sample represented the pooled contents of two culture dishes at each time point, and four independent identical experiments were performed on different days, resulting in four independent biological replicates at each time point. For the steady-state levels of probe set–identified transcripts from the microarray analyses, we determined significant differences using one-way analysis of variance (ANOVA). For the real-time PCR results, significant differences in mRNA levels were determined by two-tailed unpaired Student’s *t* tests. For the decay rate comparisons, significant differences were determined using EDGE, version 1.1.291 (http://www.genomine.org/edge/) (Storey et al. 2007) and ORIOGEN, version 4.02 (http://www.niehs.nih.gov/research/resources/software/biostatistics/oriogen/) (Peddada et al. 2005) software. Both EDGE and ORIOGEN have the capability to perform significance analysis on time-course data, and both have options for identifying genes that show different expression over time between two biological conditions.

## Results

*Cytotoxicity of sodium arsenite in BJ cells*. To determine a noncytotoxic concentration of sodium arsenite in these cells, we incubated BJ cells (at ~ 80% confluence) in normal growth medium for 24 hr and then added sodium arsenite at concentrations ranging from 0.001 to 1,000 μM; cell viability was determined by propidium iodide staining and flow cytometric analysis (see Supplemental Material, Figure S1). After 24 hr of arsenite exposure, the percentages of viable cells were 98.7 ± 0.3% (mean ± SD of 4–5 samples), 98.5 ± 0.8%, and 95.0 ± 2.3% for arsenite concentrations at 0.1, 1, and 10 μM, respectively, with rapidly increasing cytotoxicity at higher concentrations. We therefore chose 1 μM as the highest clearly noncytotoxic concentration. Similar concentrations have been used in the literature, for example, in human keratinocytes and fibroblasts (Burnichon et al. 2003; Hu et al. 2002; Snow et al. 2005).

*Effect of sodium arsenite on steady-state transcript levels in BJ cells*. We then performed a genome-wide analysis of steady-state mRNA levels in human diploid fibroblasts after 24 hr treatment with 1 μM sodium arsenite. Although there are limitations of microarray technology that have been widely discussed (Bradford et al. 2010; Malone and Oliver 2011), the technique allows for the initial screening of most of the known transcriptome; ideally, positive results are confirmed by more quantitative techniques. When referring to the microarray data, we refer to the normalized results for a specific probe set by the term “probe set–identified transcripts,” whereas we use simply “transcript” or “mRNA” when referring to the results of real-time RT-PCR analyses. Using Affymetrix microarray analysis, we identified 186 probe set–identified transcripts that were significantly up-regulated in arsenite-treated cells compared with vehicle-treated cells (*p* < 0.05), 54 (29%) of which were increased more than 2-fold after arsenic stimulation (Table 1). The greatest fold induction (68-fold) was seen with a probe set identifying *HMOX1*, the gene that encodes heme oxygenase-1, the rate-limiting enzyme in heme catabolism.

**Table 1:** Probe set–identified transcripts up-regulated by ≥ 2-fold in response to 24‑hr arsenite exposure.

Sequence code	Accession no.^*a*^	Sequence	Description	Arsenite/vehicle control	
				Fold change time 0	ANOVA *p*-value time 0
203665_at	NM_002133	*HMOX1*	Heme oxygenase (decycling) 1	68.36	1.45 × 10^–13^
209699_x_at	U05598	*AKR1C2*	Aldo-keto reductase family 1, member C2	15.74	2.92 × 10^–11^
204151_x_at	NM_001353	*AKR1C1*	Aldo-keto reductase family 1, member C1	11.99	1.45 × 10^–13^
207528_s_at	NM_014331	*SLC7A11*	Solute carrier family 7, member 11	7.42	1.45 × 10^–13^
241418_at	AI819386	*LOC344887*		7.30	1.45 × 10^–13^
213112_s_at	N30649	*SQSTM1*	UPF0544 protein	6.85	1.45 × 10^–13^
206561_s_at	NM_020299	*AKR1B10*	Aldo-keto reductase family 1, member B10	5.92	5.61 × 10^–9^
207469_s_at	NM_003662	*PIR*	Pirin (iron-binding nuclear protein)	5.39	1.45 × 10^–13^
234986_at	AA630626	*GCLM*	Glutamate-cysteine ligase, modifier subunit	5.19	1.45 × 10^–13^
219926_at	NM_022361	*POPDC3*	Popeye domain containing 3	4.71	1.71 × 10^–8^
204341_at	NM_006470	*TRIM16*	Tripartite motif-containing 16	3.51	4.86 × 10^–12^
211071_s_at	BC006471	*MLLT11*	Myeloid/lymphoid or mixed-lineage leukemia	3.36	2.21 × 10^–10^
221064_s_at	NM_023076	*UNKL*	Unkempt homolog-like	3.34	1 × 10^–5 ^
219902_at	NM_017614	*BHMT2*	Betaine-homocysteine methyltransferase 2	3.25	1.45 × 10^–13^
225252_at	AL121758	*SRXN1*	Sulfiredoxin 1 homolog	3.12	1.45 × 10^–13^
201468_s_at	NM_000903	*NQO1*	NAD(P)H dehydrogenase, quinone 1	2.95	1.45 × 10^–13^
228580_at	AI828007	*HTRA3*	Serine protease HTRA3 isoform X1	2.92	1.45 × 10^–13^
224461_s_at	BC006121	*MGC13000*	Apoptosis-inducing factor 2	2.92	4.05 × 10^–9^
218416_s_at	AW149696	*SLC48A1*	Solute carrier family 48 (heme)	2.90	2.7 × 10^–6^
219475_at	NM_013370	*OSGIN1*	Oxidative stress induced growth inhibitor 1	2.86	3 × 10^–5 ^
207850_at	NM_002090	*CXCL3*	Chemokine (C-X-C motif) ligand 3	2.78	7.4 × 10^–4 ^
208161_s_at	NM_020037	*ABCC3*	Canalicular multispecific organic anion transporter 2 isoform 1	2.76	4.54 × 10^–3 ^
212314_at	AB018289	*KIAA0746*	KIAA0746 protein	2.75	1.55 × 10^–12^
214211_at	AA083483	*FTH1*	Ferritin heavy chain	2.71	1.45 × 10^–13^
205633_s_at	NM_000688	*ALAS1*	Aminolevulinate, delta-, synthase 1	2.68	1.45 × 10^–13^
235548_at	BG326592	*APCDD1L*	Adenomatosis polyposis coli down-regulated 1-like	2.50	5 × 10^–5 ^
239067_s_at	AI360417	*PANX2*	Pannexin 2	2.47	1.7 × 10^–3 ^
209875_s_at	M83248	*SPP1*	Secreted phosphoprotein 1	2.44	4.86 × 10^–11^
207180_s_at	NM_006410	*HTATIP2*	HIV-1 Tat interactive protein 2, 30 kDa	2.41	1.45 × 10^–13^
205608_s_at	U83508	*ANGPT1*	Angiopoietin 1	2.39	4.9 × 10^–4 ^
202017_at	NM_000120	*EPHX1*	Epoxide hydrolase 1, microsomal	2.30	9.25 × 10^–11^
203192_at	NM_005689	*ABCB6*	ATP-binding cassette, sub-family B, member 6	2.24	4.1 × 10^–4 ^
201118_at	NM_002631	*PGD*	Phosphogluconate dehydrogenase	2.21	1.45 × 10^–13^
228955_at	AL041761			2.18	6.14 × 10^–8^
204059_s_at	NM_002395	*ME1*	Malic enzyme 1, NADP(+)-dependent, cytosolic	2.18	1.45 × 10^–13^
202275_at	NM_000402	*G6PD*	Glucose-6-phosphate dehydrogenase	2.09	3.96 × 10^–13^
1563884_at	AK074255			2.05	6.6 × 10^–3 ^
228205_at	AU152969	*TKT*	Transketolase, transcript variant X1	2.04	1.7 × 10^–4 ^
228937_at	AI659800	*C13orf31*		2.03	2.73 × 10^–10^
217359_s_at	M22094	*NCAM1*	Neural cell adhesion molecule 1	2.00	5.17 × 10^–3 ^
Blanks under “Sequence” and “Description” indicate that no gene name or function has been ascribed to those transcripts. Many of the transcripts represented here were identified by multiple probe sets, but only one probe set–identified transcript is shown for each gene. ^***a***^GenBank accession numbers (http://www.ncbi.nlm.nih.gov/genbank/).					

Similarly, 167 probe set–identified transcripts were significantly down-regulated. Those changed by ≥ 2-fold are shown in Table 2. The most down-regulated gene was *TNFRSF19,* which encodes tumor necrosis factor receptor superfamily member 19, thought to be responsible for regulating several immediate-response molecules such as NF-κB, RhoA, and Jun (Eby et al. 2000; Mi 2008).

**Table 2:** Probe set–identified transcripts down-regulated by ≥ 2-fold in response to 24‑hr arsenite exposure.

Sequence code	Accession no.^*a*^	Sequence	Description	Arsenite/vehicle control	
				Fold change time 0	ANOVA *p*-value time 0
227812_at	BF432648	*TNFRSF19*	Tumor necrosis factor receptor superfamily, member 19	–2.87	3.35 × 10^–10^
1554685_a_at	BC020256	*KIAA1199*	Protein KIAA1199 precursor	–2.77	1.2 × 10^–9^
1559315_s_at	AK054607	*LOC144481*		–2.72	3.59 × 10^–12^
209602_s_at	AI796169	*GATA3*	GATA binding protein 3	–2.48	6.07 × 10^–3^
206528_at	NM_004621	*TRPC6*	Transient receptor potential cation channel, subfamily C, member 6	–2.47	5.89 × 10^–3^
227488_at	AV728999	*MGC16121*		–2.47	5.6 × 10^–4 ^
201194_at	NM_003009	*SEPW1*	Selenoprotein W, 1	–2.45	1.45 × 10^–13^
205479_s_at	NM_002658	*PLAU*	Plasminogen activator, urokinase	–2.40	3.4 × 10^–4 ^
1555997_s_at	BM128432	*IGFBP5*	Insulin-like growth factor binding protein 5	–2.24	5.63 × 10^–8^
228509_at	BE549786	*SPHKAP*	SPHK1 interactor, AKAP domain containing	–2.21	1.32 × 10^–3 ^
228335_at	AW264204	*CLDN11*	Claudin 11	–2.15	1.45 × 10^–13^
203372_s_at	AB004903	*SOCS2*	Suppressor of cytokine signaling 2	–2.12	6.24 × 10^–10^
204337_at	AL514445	*RGS4*	Regulator of G-protein signaling 4	–2.08	6.0 × 10^–5 ^
216598_s_at	S69738	*CCL2*	Chemokine (C-C motif) ligand 2	–2.06	8.8 × 10^–4 ^
228329_at	AA700440	*DAB1*	Disabled-1	–2.02	6.37 × 10^–7^
229357_at	BF060767	*ADAMTS5*	Zinc metalloprotease	–2.01	1.04 × 10^–11^
203153_at	NM_001548	*IFIT1*	Interferon-induced protein with tetratricopeptide repeats 1	–2.00	4.2 × 10^–4 ^
Blanks under “Sequence” and “Description” indicate that no gene name or function has been ascribed to those transcripts. Many of the transcripts represented here were identified by multiple probe sets, but only one probe set–identified transcript is shown for each gene. ^***a***^GenBank accession numbers (http://www.ncbi.nlm.nih.gov/genbank/).					

Several previous studies have used microrarray analyses to investigate the effects of arsenite on gene expression in human and mouse fibroblasts (Maeshima et al. 2009; Newman et al. 2008; Poonepalli et al. 2005; Yih et al. 2002; Yu et al. 2008). Of these, we were able to compare our expression patterns in human cells only with those of Yu et al. (2008) in mouse fibroblasts, which were exposed to 5 μM arsenite for 24 hr. The only overlaps with our up-regulated genes were *Hmox1*, *Gclm*, and *Nqo1*, which Yu et al. (2008) reported were increased at 4.2-, 2.2-, and 2.1-fold, respectively, and the overlaps with our down-regulated genes were *Sepw1* and *Ifit1*, which were down-regulated by 1.9- and 2.6-fold, respectively.

Overall, only 353 of the 54,613 probe sets analyzed (0.65%) demonstrated steady-state probe set–identified transcript levels that were significantly altered after 24 hr of treatment with sodium arsenite. Analysis of these data by IPA is summarized in Supplemental Material, Tables S2–S5; these results highlight effects on many pathways, including NRF2-mediated oxidative stress response, the pentose phosphate pathway, vitamin C transport, heme degradation, methylglyoxal degradation, heme biosynthesis, and others.

*Effect of sodium arsenite on mRNA decay rates*. To determine whether the observed changes in probe set–identified transcript levels were due, at least in part, to changes in their decay rates, we performed microarray analysis on RNA samples isolated from control or arsenite-treated cells at hourly intervals for 4 hr after the addition of actinomycin D; little or no actinomycin D–induced cytotoxicity is thought to occur before 4 hr, but longer exposures can complicate interpretation of results (Sawicki and Godman 1971; Valeriote et al. 1973). The cells had been exposed to control conditions or 1 μM arsenite for 24 hr prior to the actinomycin D treatment. For convenience of presentation, we converted the microarray data for arsenite and vehicle treatment groups to the percentage of their respective abundance at time 0, which was set as 100%.

We first identified a set of probe set–identified transcripts that were likely to be analyzable by this method, that is, those that decayed at rates fast enough that we might expect to see differences in this relatively short-term experiment. Accordingly, of the 54,613 original probe sets, only 4,992 (9.1%) decayed by ≥ 25% after 4 hr in the control cells, equating to approximate half-lives of ≤ 8 hr. The slower decay rates of the remaining 90% meant that we were unlikely to be able to detect decay rate differences for them using this method of analysis. We therefore focused the rest of our analysis on the 4,992 probe set–identified transcripts that decayed by ≥ 25% in 4 hr.

**Figure 1:**
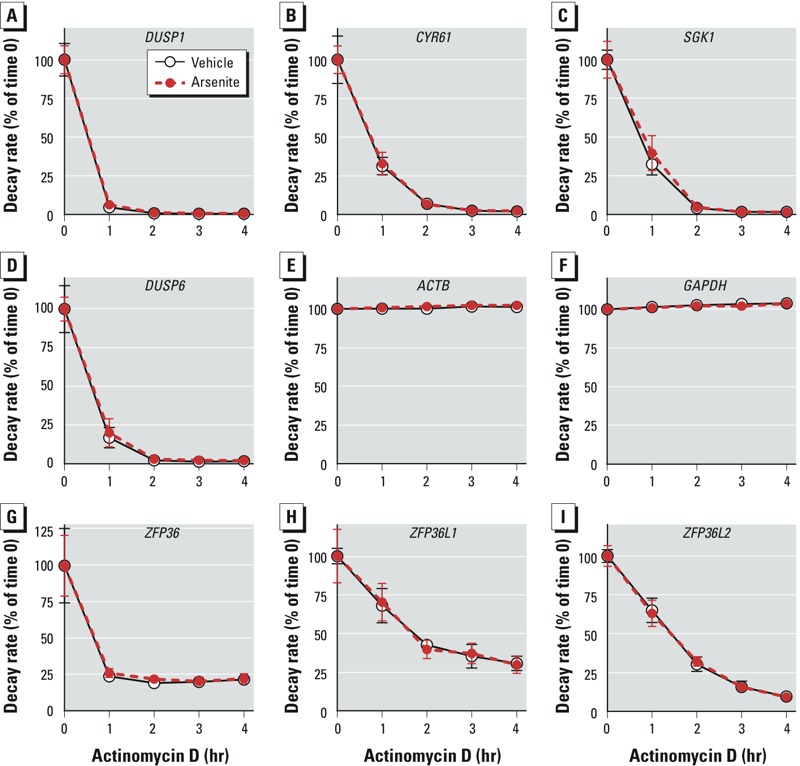
Decay rates of selected probe set–identified transcripts measured by microarray in cells treated with vehicle or arsenite for 24 hr and then treated with actinomycin D. Probe set–identified transcript levels were determined before and at 1 hr intervals after actinomycin D treatment. The starting levels of each probe set–identified transcript after 24 hr of treatment but before actinomycin D were set at 100%, and data are expressed as mean percentages (± SD) of the average starting value (*n* = 4 biological replicates per group). Decay curves for vehicle- and arsenite- treated cells are shown for the four most rapidly decaying probe set–identified transcripts (*A*–*D*); two expected to be stable under these conditions, encoding GAPDH and ACTB (*E*,*F*); and three encoding the TTP family members expressed in human cells, *ZFP36* (TTP; *G*), *ZFP36L1* (*H*), and *ZFP36L2* (*I*). In these examples, there were no differences between the decay rates between arsenite- and vehicle-treated cells. The Affymetrix probe set identifiers for the transcripts shown in this figure are as follows: *DUSP1*, 201041_s_at; *CYR61*, 210764_s_at; *SGK1*, 201739_at; *DUSP6*, 208892_s_at; *ACTB*, 224594_x_at; *GAPDH*, 213453_x_at; *ZFP36*, 201531_at; *ZFP36L1*, 211962_s_at; and *ZFP36L2*, 201368_at.

To confirm the effectiveness of the actinomycin D treatment under these experimental conditions, we examined comparative decay curves for several of the most rapidly decaying probe set–identified transcripts, as well as those expected to be stable in most cell types, such as those encoding ACTB and GAPDH. Figure 1A–D shows the four probe set–identified transcripts that decayed most rapidly under these conditions. Figure 1 also includes data for two probe sets representing transcripts expected to be stable, *ACTB* and *GAPDH* mRNAs (Figure 1E,F), as well as transcripts encoding the three members of the tristetraprolin (TTP) mRNA-destabilizing protein family that are expressed in humans and are known to participate in mRNA decay mediated by adenylate-uridylate-rich elements (Figure 1G–I). In these examples, we observed no apparent differences between decay rates in the arsenite and control samples, with decay rates being essentially superimposable in all cases. The observed very rapid decay of some probe set–identified transcripts, the lack of decay of known stable transcripts, the relatively narrow confidence limits, and the essentially identical results in the arsenite and control samples all support the effectiveness of the microarray screen and of actinomycin D as a rapidly acting and effective transcription inhibitor under these conditions.

**Figure 2:**
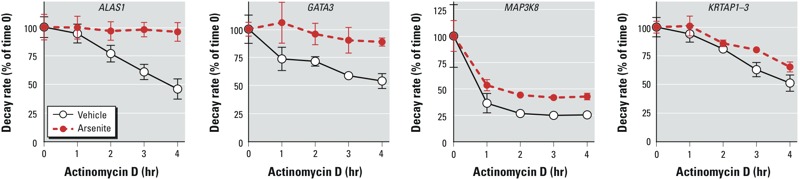
Decay rates of four probe set–identified transcripts for which the steady-state levels and decay rates were significantly different in arsenite-treated cells compared with control. Cells were treated with vehicle or arsenite for 24 hr and then treated with actinomycin D. Probe set–identified transcript levels were measured by microarray before and at 1 hr intervals after actinomycin D treatment. The starting levels of each probe set–identified transcript after 24 hr of treatment but before actinomycin D were set at 100%, and the data are expressed as mean percentages (± SD) of the average starting value (*n* = 4 biological replicates per group). In all cases, the decay curves were significantly different between control and arsenite-treated cells by both the ORIOGEN and EDGE methods (*p* < 0.05). The Affymetrix probe set identifiers for the transcripts shown are as follows: *ALAS1*, 205633_s_at; *GATA3*, 209602_s_at; *MAP3K8*, 205027_s_at; and *KRTAP1‑3*, 234880_x_at.

Of the 4,992 probe set–identified transcripts that decayed by ≥ 25% after 4 hr in the control cells, 70 were were significantly different in the steady state. Of these, only 5 probe sets (for four transcripts) had significant EDGE and ORIOGEN *p*-values (*p* < 0.05) for the differences between the control and arsenite decay curves. For these 5, arsenite increased stability in every case. The mRNAs identified by these probe sets were those encoding ALAS1, GATA3 (twice), MAP3K8, and KRTAP1 (Figure 2). Of the probe set–identified transcripts shown in Figure 2, only *ALAS1* and *GATA3* mRNAs were on the list of transcripts that were up- or down-regulated by ≥ 2-fold in response to arsenite. In the case of *ALAS1* mRNA, the change in decay induced by arsenite (stabilization) was in the same direction as the steady-state increase of 2.68-fold caused by arsenite after 24 hr (Table 1); for *GATA3* mRNA, the apparent stabilization in the presence of arsenite occurred despite the effect of arsenite to decrease steady-state levels by 2.48-fold (Table 2).

**Figure 3:**
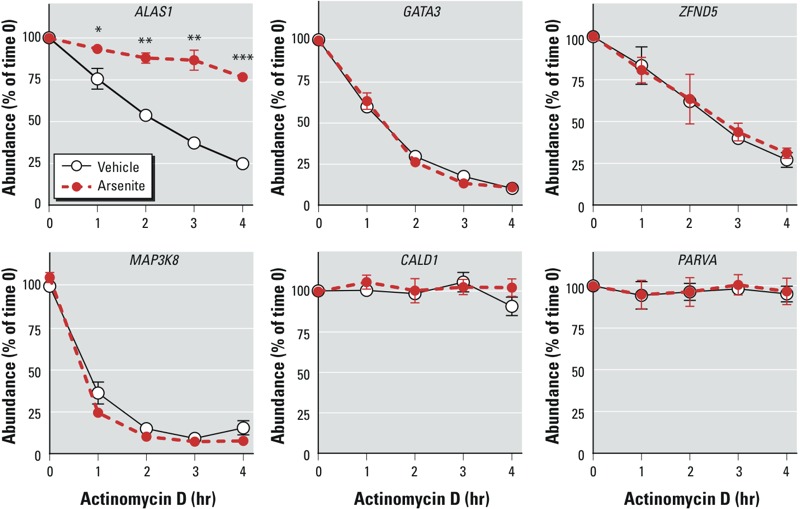
Effect of arsenite on the stability of *ALAS1*, *GATA3, ZFND5, MAP3K8, CALD1, *and *PARVA *mRNA in cells treated with vehicle or arsenite for 24 hr and then treated with actinomycin D for 1–4 hr, as determined by real-time RT-PCR. The apparent arsenite-induced stabilization of *ALAS1* mRNA seen in the microarray analyses was confirmed; however, the differences in decay rates suggested by microarray for *GATA3* and *MAP3KB* mRNAs shown in Figure 2 were not confirmed. Arsenite showed no effect on the decay rates of several other transcripts, including one with an intermediate half-life (*ZFAND5*) and two relatively stable transcripts (*CALD1* and *PARVA*) during the 4-hr time course. Relative transcript abundance was calculated as a fraction of transcript abundance at time 0, before the addition of actinomycin D (set at 100%). Data are expressed as mean ± SE of four independent experiments. *p < 0.05. **p < 0.01. ***p < 0.001.

To confirm the changes suggested by the microarray data for the *ALAS1* and *GATA3* mRNAs, we performed real-time RT-PCR on the same mRNA samples, using primers designed specifically for these transcripts [see “Real-time reverse transcription polymerase chain reaction (RT-PCR)”]. The real-time RT-PCR analysis confirmed the highly significant differences between the decay curves for *ALAS1* mRNA observed in the microarray data (Figure 3) and confirmed that this transcript was greatly stabilized in the presence of arsenite. In the case of *GATA3*, the real-time RT-PCR data did not confirm a difference in the decay rates between the arsenite and control samples (Figure 3). Real-time RT-PCR analysis of some of the other transcripts identified in the initial screen failed to demonstrate differences in decay rates for *ZFAND5*, *MAP3K8*, *CALD1,* and *PARVA* mRNAs (Figure 3).

We then investigated the remaining probe set–identified transcripts that decayed > 25% in the control cells at 4 hr but did not exhibit significant differences in steady-state mRNA levels after 24 hr of control or arsenite treatment. Of the original set of 4,992 probe set–identified transcripts that decayed > 25% after 4 hr of actinomycin D treatment, 340 exhibited significant (*p* < 0.05) differences in both EDGE and ORIOGEN *p*-values for the decay curves. After removing the transcripts discussed above that had significant steady-state changes, uncharacterized genes, and duplicates, 162 probe set–identified transcripts remained, of which 35 were significantly destabilized by arsenite; the remaining 127 were stabilized. Of these 162, 49 had EDGE and ORIOGEN *p*-values that were significant at the *p* < 0.01 level for both tests. Supplemental Material, Table S6, provides a list of these 49 probe set–identified transcripts along with their average percentage decreases at 4 hr compared with time 0 and the ORIOGEN and EDGE *p*-values for the decay curves. Examples of probe set–identified transcripts stabilized by arsenite are shown in Supplemental Material, Figure S2, and examples of those destabilized by arsenite are shown in Supplemental Material, Figure S3. It should be noted that the differences between the decay curves for these transcripts were deemed highly significant (*p* < 0.01) by both tests, resulting in a fairly stringent selection. Of the 4,992 probe set–identified transcripts that decayed by > 25% in 4 hr in the control cells, 466 were considered significant by ORIOGEN analysis (*p* < 0.05) but not by EDGE analysis. Similarly, 98 of these probe set–identified transcripts were deemed significant by EDGE analysis (*p* < 0.05) but not by ORIOGEN analysis. These individual tests provide less stringency than the two together, but they may provide clues in some instances to biologically significant changes in mRNA stability.

## Discussion

The major goal of this study was to determine whether changes in mRNA stability contribute to the changes in steady-state mRNA levels seen in human diploid fibroblasts treated with sodium arsenite. In this genome-wide microarray analysis, we identified 353 probe set–identified transcripts (0.6% of 54,613) whose steady-state levels were significantly altered after 24-hr exposure to a noncytotoxic concentration of arsenite. Of these 353, only 70 decayed rapidly enough after actinomycin D treatment (approximate half-lives of < 8 hr) to make comparisons between the decay rates feasible by these techniques. Of these 70, only 4 transcripts exhibited differences in decay rates between control and arsenite-treated cells, and only 1 probe set, corresponding to the transcript of *ALAS1*, had a decay rate change that was in the appropriate direction to account for the steady-state mRNA levels. In this case, the steady-state transcript levels were significantly increased after arsenite treatment compared with the control (2.7-fold), and the decay curves showed highly significant transcript stabilization after arsenite treatment. This significant stability change identified by microarray was confirmed by real-time RT-PCR. These data suggest that, at least for transcripts with more rapid decay rates in fibroblasts, changes in mRNA decay rates in response to arsenite accounted for very few of the steady-state changes observed after 24 hr of treatment.

This analysis was limited by the apparent stability of most mRNAs under these experimental conditions, in which approximately 90% of probe set–identified transcripts were apparently too stable to make decay-rate comparisons feasible using these methods. This is one of the inherent difficulties with the actinomycin D method because cytotoxicity prevents longer-term experiments that would allow measurements on slower-decaying transcripts. Using recently developed techniques such as ribonucleoside labeling (Dölken 2013), it may be possible to determine turnover rates on a global scale that will also encompass these more stable transcripts.

In addition to the few changes in mRNA stability that could account, at least in part, for changes in steady-state mRNA levels after arsenite treatment, 49 probe set–identified transcripts exhibited highly significant arsenite-induced changes in stability that were not reflected in significant changes in steady-state levels after 24 hr, and only 8 of these were destabilized in the arsenite-treated cells. Many more transcripts were identified whose decay rates differed at lower rates of significance, or in only one of the two tests of significance. The fact that these transcripts did not exhibit changes in steady-state levels suggests the possibility of compensatory changes in transcription rates in these cases.

The striking stabilization of *ALAS1* mRNA observed in both microarray and real-time RT-PCR presumably contributed to its steady-state increase after arsenite treatment. *ALAS1* encodes aminolevulinate, delta-, synthase 1, a mitochondrial protein that is the rate-limiting step in heme biosynthesis. Heme has long been known to affect *ALAS1* expression at the levels of transcription, mRNA decay, mitochondrial import and export, and protein stability (Furuyama et al. 2007; Schuurmans et al. 2001). In particular, low cellular levels of heme appear to be able to both increase *ALAS1* transcription and *ALAS1* mRNA stability. Because the most dramatically up-regulated gene in arsenite-treated cells was *HMOX1*, encoding heme oxygenase-1 (the rate-limiting enzyme in heme catabolism), a plausible mechanism for the effect of arsenite to increase *ALAS1* mRNA steady-state levels and mRNA stability would be as follows: Arsenite treatment would cause massive increases in *HMOX1* expression, resulting in increases in cellular heme catabolism and decreases in heme levels; the decreased heme levels would then lead to increases in *ALAS1* transcription and mRNA stability, resulting in increased heme biosynthesis. It seems possible that this effect on heme biosynthesis could lead to known effects of arsenic exposure on exacerbations of porphyria (Tian et al. 2011) and various erythrocyte disorders (Lisiewicz 1993). Earlier studies in human fibroblasts demonstrated that the transcriptional activation of *HMOX1*, but not its mRNA stability, is the major mechanism for arsenic-induced accumulation of *HMOX1* mRNA and protein (Keyse et al. 1990). The present study confirmed that the huge increase in *HMOX1* mRNA levels seen after arsenite (68-fold increase) was not accompanied by significant changes in *HMOX1* mRNA decay rates, at least on a percentage basis. It should be noted that many previous studies have demonstrated induction of *HMOX1* after treatment of cells with various forms of arsenic (Liu et al. 2001, 2006; Rea et al. 2003; Wu et al. 2008; Yih et al. 2002).

## Conclusions

We found that arsenite modification of mRNA stability leading to changes in steady-state levels is very uncommon, at least among the most rapidly decaying 10% of transcripts under our experimental conditions. However, arsenite clearly affected *ALAS1* mRNA stability in this experimental setting, which, at least in part, contributed to the significant steady-state increase in this mRNA. This change in transcript stability presumably leads to an increase in protein, which in turn should play a role in attempting to maintain intracellular heme levels in response to the anticipated depletion caused by the massive induction of *HMOX1*. The detailed mechanism by which arsenite exerts its posttranscriptional control of *ALAS1* mRNA remains to be clarified. Further studies using different methods will be necessary to determine whether arsenite can cause changes in stability in the vast majority of fibroblast mRNAs that decay with half-lives > 8 hr.

## Supplemental Material

(501 KB) PDF
