# Supplementary material for: Global Analysis of Posttranscriptional Gene Expression in Response to Sodium Arsenite
Source: Environ Health Perspect. 2014 Nov 21;123(4):324–30. doi: 10.1289/ehp.1408626 (PMC4383576; doi:10.1289/ehp.1408626)
Supplement: (501 KB) PDF [file ehp.1408626.s001.508.pdf]

Environmental Health Perspectives doi:10.1289/ehp.1408626

Supplemental Material

Global Analysis of Posttranscriptional Gene Expression in Response to Sodium Arsenite

Lian-Qun Qiu, Sarah Abey, Shawn Harris, Ruchir Shah, Kevin E. Gerrish, and Perry J. Blackshear

Figure S1.

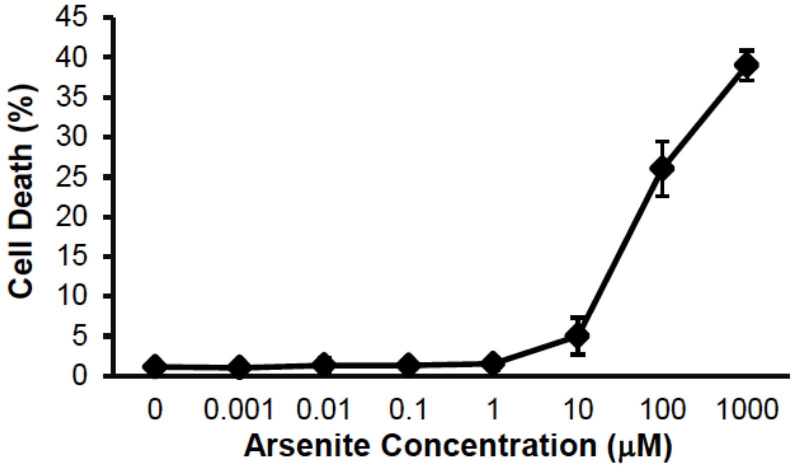

Figure S1. Cytotoxicity assay of sodium arsenite in human foreskin fibroblasts. Rates of cell death were determined using propidium iodide staining followed by flow cytometric analysis after 24 h of treatment of human BJ fibroblasts with sodium arsenite at the concentrations indicated. Results shown are the means ± SD from 2-5 experiments.

Figure S2.

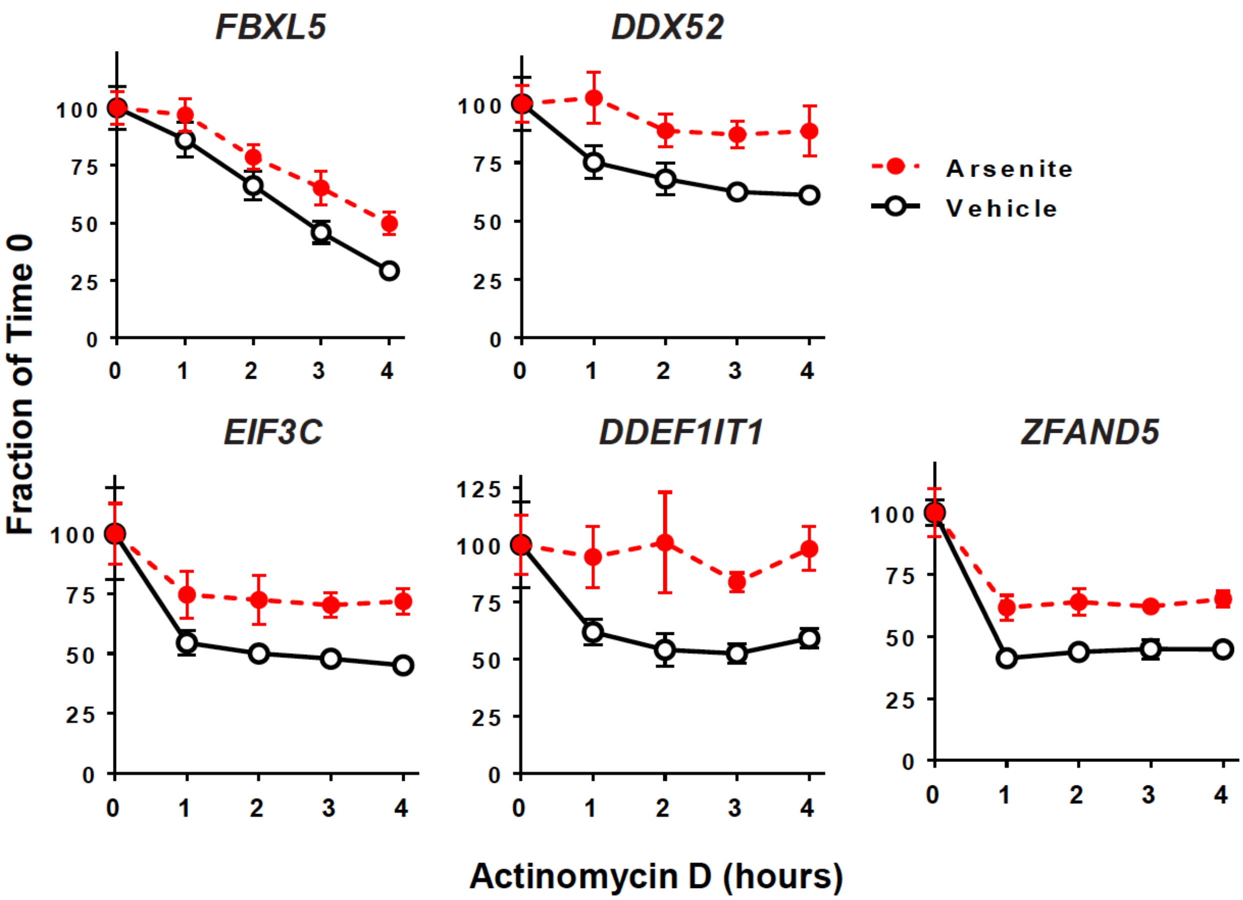

Figure S2. Decay rates of probe set-identified transcripts measured by microarray whose steady state levels after arsenite were not significantly different from control, but whose decay rates were significantly decreased by arsenite. Shown are the decay rates for five transcripts whose steady state levels were not significantly different in the control and arsenite-treated cells, but whose decay rates were significantly different by both the Oriogen and Edge methods ($P<0.05$). In these five examples, arsenite stabilized the mRNA. The Affymetrix probe set identifiers used for these transcripts were: FBXL5, 209004_s_at; DDX52, 1570415_at; EIF3C, 236700_at; DDEF1IT1, 217649_at; ZFAND5, 220694_at. See the legend to Figure 1 for details.

Figure S3.

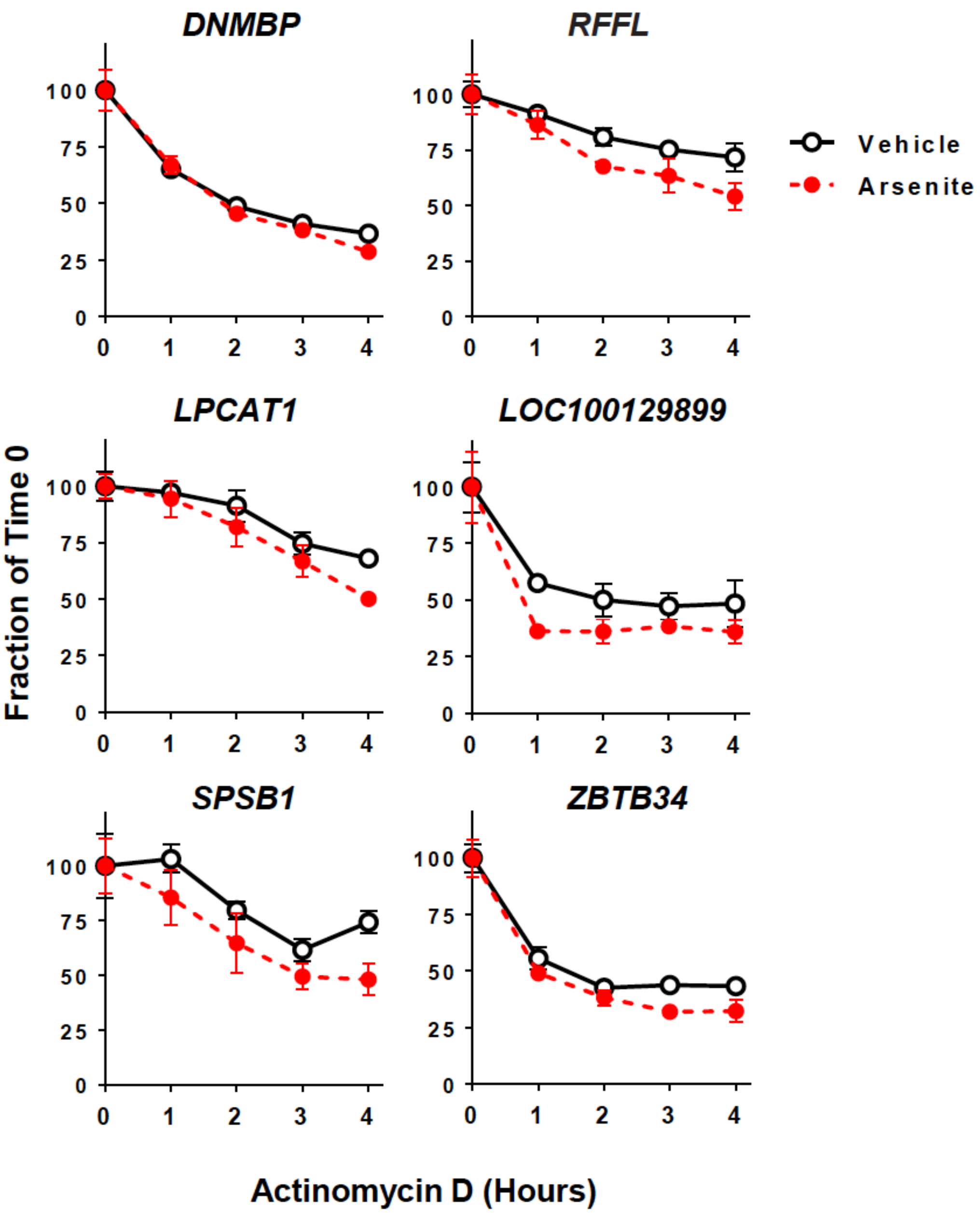

Figure S3. Decay rates of probe-set identified transcripts measured by microarray whose steady state levels after arsenite were not significantly different from control, but whose decay rates were significantly decreased by arsenite. Shown are the decay rates for six transcripts whose steady state levels were not significantly different in the control and arsenite-treated cells, but whose decay rates were significantly different by both the Oriogen and Edge methods ($P<0.05$).

The starting levels of each probe set-identified transcript after 24 h of treatment but before actinomycin D were set at 100%, and the other data are expressed as mean percentages +/- SD of that average starting value (n=4 biological replicates in each group). In these six examples, arsenite destabilized the mRNA. The Affymetrix probe set identifiers used for these transcripts were: DNMBP, 212838_at; RFFL, 228980_at; LPCAT1, 201818_at; LOC100129899, 238771_at; SPSB1, 219677_at; and ZBTB34, 227111_at. See the legend to Figure 1 for details.

Table S1. Primers used in the study.

Gene	GenBank Accession #	Amplicon length (bp)	Primer Sequences
ALAS1	NM_000688.4	120	Forward: 5'-CAACCCTCTTCACCCTGGCTAA-3'<br>Reverse: 5'-GGCGGAAGATGTACTTTGGCA-3'
ATP5C1	NM_001001973.1	148	Forward: 5’-TTTGGAGATGCGTCAGTCATTG-3’<br>Reverse: 5’-CACTTGCAACGGTATTAAGGGAA-3’
BACTIN	NM_001101.3	137	Forward: 5'-AACTGGGACGACATGGAGAAA-3'<br>Reverse: 5'-ATGATCTGGGTCATCTTCTCGC-3'
C3orf34	NM_032898.3	92	Forward: 5'-AGAATAATCCGCGACACAAGAGTT-3'<br>Reverse: 5'-CCCCGACAAGTAACCTCGTAAA-3'
CALU	NM_001130674.2	152	Forward: 5’-TGACTTATGGCACTTACCTGGATGA-3’<br>Reverse: 5’-AGGGTGCAGGAAAGCTGTGAA-3’
CALD1	NM_033138.2	158	Forward: 5'-AGCAGGCACACCAAATAAGGAA-3'<br>Reverse: 5'-CCCAGAGGTTCCGCTTGCT-3'
CTNNB1	NM_001098209.1	153	Forward: 5’-TGACCAGCCGACACCAAGAA-3’<br>Reverse: 5’-CGGGACAAAGGGCAAGATTT-3’
EIF5A	NM_001143760.1	155	Forward: 5’-TCAGCCACCTTCCCAATGC-3’<br>Reverse: 5’-CCAGTAAAGATGTCAATACCAACCA-3’
GATA3	NM_001002295.1	95	Forward: 5'-CCTCATTAAGCCCAAGCGAA-3'<br>Reverse: 5'-TCCTCCTCCAGAGTGTGGTTGT-3'
GAPDH	NM_002046.3	105	Forward: 5'-CACCCACTCCTCCACCTTTGA-3'<br>Reverse: 5'-CTGTTGCTGTAGCCAAATTCGTT-3'
GCNT4	NM_016591.2	117	Forward: 5'-GAGCACTTTTGGGCTACCTTGA-3'<br>Reverse: 5'-ATAGTAATTCCACTTGACAAGGCGA-3'
GRPEL2	NM_152407.3	142	Forward: 5’-GGAAGACGCCAAGATATTTGGAAT-3’<br>Reverse: 5’-TCGGAAGACCTTCTCCAGAGTGA -3’
GSK3B	NM_001146156.1	163	Forward: 5’-TCACTGTAACATAGTCCGATTGCGT-3’<br>Reverse: 5’-CTTGACATAAATCACAGGGAGCGT-3’
hLMO7	NM_005358.5	108	Forward: 5’-TTCGGTGGTTCCTGATCTTCC-3’<br>Reverse: 5’-TCCTGCTCCTTCTGCCACCT-3’
LRRFIP1	NM_001137550.1	169	Forward: 5’-TGGATACCAAGGTCCTACCAAGAT-3’<br>Reverse: 5’-GCCAATCTTCTGTCTCTCCTCCA-3’
MAP3K8	NM_005204.2	127	Forward: 5’-AAGAGGCTGCTGAGTAGGAAGGA-3’<br>Reverse: 5’-GAGCGCCGAGGTCGATGTA-3’
NAMPT	NM_005746.2	131	Forward: 5’-CCAAGAGACTGCTGGCATAGGA-3’<br>Reverse: 5’-CTGGAACAGAATAGCCTGGAACA-3’
NRG1	NM_001159995.1	115	Forward: 5’-CTGTCACCCAGACTCCTAGCCA-3’<br>Reverse: 5’-GGGCTGCTGTGCCTACTGTTT-3’
PARVA	NM_018222.3	165	Forward: 5'-GGAATCCTCCAGTCTCGGCA-3'<br>Reverse: 5'-ATTCAGGTGCTTGTTCACGAAAGT-3'
PEX13	NM_002618.3	191	Forward: 5’-CTGAGAATGAAGACCTCTGGGCA -3’<br>Reverse: 5’-TGCCCAGTTGATGCTGTCTGTTAC-3’
PHF8	NM_015107.2	142	Forward: 5'-AGGCGACCCTGATAATAAGACCA-3'<br>Reverse: 5'-TCCACCATGTCCTCATCTGTTGTA-3'
PPARA	NM_001001928.2	138	Forward: 5’-CAAGAAATGGGAAACATCCAAGAG-3’<br>Reverse: 5’-TGGTGAAAGCGTGTCCGTGA-3’
SLAIN2	NM_020846.1	194	Forward: 5’-GCCATCCCACAGATTTACAGACAA-3’<br>Reverse: 5’-GCCTGGAGAAGGTATGGCTGAA-3’
STANNIN	NM_003498.4	124	Forward: 5'-CCCAGCACTGACCATGTCTATTAT-3'<br>Reverse: 5'-CCGCAGGTAGCACCAGCA-3'
ZCCHC6	NM_024617.3	143	Forward: 5’-TGAGCAGGATGGAGACTTGGAA-3’<br>Reverse: 5’-TAGGGTAGTTTCGTGGTCGCC-3’
ZFAND5	NM_001102420.1	135	Forward: 5’-CTCAGCCCAGTCCATCAGTTTCT-3’<br>Reverse: 5’-ATCGGCAGTCAAACCCTGTAAGA-3’

Table S2. Top networks based on IPA analysis of up-regulated probe-set identified transcripts.

Rank	Associated Network Functions	Score
1	Small molecule biochemistry, carbohydrate metabolism, nucleic acid metabolism	57
2	Organismal injury and abnormalities, renal and urological disease, cell death and survival	40
3	Small molecule biochemistry, cellular development, cellular growth and proliferation	40
4	Lipid metabolism, small molecule biochemistry, cellular assembly and organization	26
5	Cancer, cell death and survival, cellular development	21

Table S3. Top biological functions, canonical pathways, upstream regulators, and tox lists based on IPA analysis of up-regulated probe-set identified transcripts.

Name	p-Value	# Molecules	Ratio	Predicted Activation State
**Top biological functions**				
Diseases and Disorders				
Cancer	1.37E-11 - 7.52E-03	88		
Gastrointestinal Disease	3.08E-11 - 7.52E-03	48		
Hepatic System Disease	1.31E-06 - 3.30E-03	19		
Organismal Injury and Abnormalities	1.53E-06 - 7.52E-03	27		
Renal and Urological Disease	4.64E-06 - 7.52E-03	21		
Molecular and Cellular Functions				
Cell Death and Survival	9.38E-09 - 7.52E-03	64		
Carbohydrate Metabolism	3.74E-07 - 7.52E-03	23		
Nucleic Acid Metabolism	3.74E-07 - 7.52E-03	15		
Small Molecule Biochemistry	2.07E-06 - 7.52E-03	41		
Cell Morphology	5.62E-05 - 7.52E-03	14		
Physiological System Development and Function				
Organ Morphology	1.09E-05 - 7.52E-03	19		
Renal and Urological System Development and Function	1.09E-05 - 7.52E-03	12		
Cardiovascular System Development and Function	5.62E-05 - 7.52E-03	15		
Embryonic Development	5.62E-05 - 7.52E-03	13		
Hepatic System Development and Function	5.62E-05 - 5.85E-03	6		
**Top Canonical Pathways**				
NRF2-mediated Oxidative Stress Response	8.1E-11		14/192 (0.073)	
Pentose Phosphate Pathway	9.69E-07		4/23 (0.174)	
Vitamin-C Transport	1.43E-04		3/22 (0.136)	
Methylglyoxal Degradation III	2.17E-04		3/23 (0.13)	
Heme Degradation	3.34E-04		2/11 (0.182)	
**Top Upstream Regulators**				
NFE2L2	8.40E-25			Activated
tert-butyl-hydroquinone	3.06E-18			Activated
arsenic trioxide	2.11E-17			Activated
2-cyano-3 12-dioxoolean-1 9-dien-28-oic acid	3.18E-17			Activated
BACH1	3.97E-15			Inhibited
**Top Tox Lists**				
NRF2-mediated Oxidative Stress Response	2.56E-09		14/234 (0.06)	
Protection from Hypoxia-induced Renal Ischemic Injury (Rat)	1.65E-06		3/4 (0.75)	
Long-term Renal Injury Anti-oxidative Response Panel (Rat)	8.62E-06		4/18 (0.222)	
Glutathione Depletion - CYP Induction and Reactive Metabolites	8.71E-05		3/12 (0.25)	

Table S4. IPA analysis of down-regulated probe-set identified transcripts.

Rank	Associated Network Functions	Score
1	Cell death and survival, embryonic development, cellular growth and proliferation	48
2	Nervous system development and function, organ morphology, antigen presentation	30
3	Cardiovascular disease, cardiovascular system development and function, lymphoid tissue structure and development	28
4	Cancer, developmental disorder, hereditary disorder	27
5	Cell death and survival, cellular assembly and organization, cellular compromise	24

Table S5. IPA analysis of down-regulated probe-set identified transcripts.

Name	p-Value	# Molecules	Ratio	Predicted Activation State
**Diseases and Disorders**				
Cancer	1.81E-09 - 6.30E-03	83		
Gastrointestinal Disease	1.95E-09 - 6.30E-03	44		
Reproductive System Disease	7.27E-08 - 6.30E-03	41		
Developmental Disorder	2.63E-06 - 6.30E-03	27		
Skeletal and Muscular Disorders	3.45E-06 - 6.30E-03	21		
**Molecular and Cellular Functions**				
Cellular Growth and Proliferation	4.73E-09 - 6.30E-03	59		
Cellular Development	3.20E-07 - 6.30E-03	51		
Cell Death and Survival	2.35E-06 - 6.30E-03	47		
Cellular Movement	3.94E-06 - 6.30E-03	37		
Cell Morphology	1.17E-04 - 6.30E-03	36		
**Physiological System Development and Function**				
Cardiovascular System Development and Function	8.42E-08 - 6.30E-03	32		
Lymphoid Tissue Structure and Development	8.42E-08 - 6.30E-03	14		
Embryonic Development	1.43E-05 - 6.30E-03	40		
Organ Development	1.43E-05 - 6.30E-03	36		
Organismal Development	1.43E-05 - 6.30E-03	49		
**Top Canonical Pathways**				
cAMP-mediated signaling	4.85E-04	7/226 (0.031)	7/226 (0.031)	
G-Protein Coupled Receptor Signaling	1.23E-03	7/275 (0.025)	7/275 (0.025)	
Interferon Signaling	1.26E-03	3/36 (0.083)	3/36 (0.083)	
Cellular Effects of Sildenafil (Viagra)	8.98E-03	4/149 (0.027)	4/149 (0.027)	
Hepatic Fibrosis / Hepatic Stellate Cell Activation	1.16E-02	4/146 (0.027)	4/146 (0.027)	
**Top Upstream Regulators**				
TNF	1.18E-10			Inhibited
Cg	3.60E-10			Inhibited
TGFB1	7.74E-09			Inhibited
Tretinoin	5.29E-08			Inhibited
beta-estradiol	1.02E-07			Inhibited
**Top Tox Lists**				
Cardiac Hypertrophy	6.29E-05		10/344 (0.029)	
Increases Heart Failure	1.85E-04		3/18 (0.167)	
Renal Necrosis/Cell Death	6.61E-04		10/461 (0.022)	
Reversible Glomerulonephritis Biomarker Panel (Rat)	1.25E-02		2/27 (0.074)	
Persistent Renal Ischemia-Reperfusion Injury (Mouse)	1.52E-02		2/30 (0.067)	

Table S6. Transcripts with changes in decay rates but not steady state levels in response to arsenite.

Affy number	Gene	Protein	RefSeq mRNA	ORIOGEN p-Value	Edge p-Value	Arsenite effect*	Control 4h avg (%)	Arsenite 4h avg (%)
1555436_a_at	*AFF4*	AF4/FMR2 family, member 4	NM_014423	0.0008	0.0023	s	72.6	96.8
226095_s_at	*ATXN1L*	ataxin 1-like	NM_001137675	0.0066	0.0020	d	61.8	54.7
215199_at	*CALD1*	caldesmon 1	NM_004342	0.0001	0.000003	s	30.2	64.5
1565868_at	*CD44*	CD44 molecule (Indian blood group)	NM_000610	0.0047	0.0029	s	53.9	73.3
233109_at	*COL12A1*	Collagen, type XII, alpha 1	NM_004370	0.0001	0.0004	s	27.5	46.1
220694_at	*DDEF1IT1*	DDEF1 intronic transcript 1 (non-protein coding)	NR_002765	0.0016	0.0001	s	59.1	98.3
1570415_at	*DDX52*	DEAD (Asp-Glu-Ala-Asp) box polypeptide 52	NM_007010	0.0022	0.0005	s	61.2	88.4
223662_x_at	*DDX59*	DEAD (Asp-Glu-Ala-Asp) box polypeptide 59	NM_001031725	0.0010	0.0031	s	65.1	73.8
212838_at	*DNMBP*	dynamin binding protein	NM_015221	0.0001	0.00007	d	36.7	28.5
236649_at	*DTWD1*	DTW domain containing 1	NM_001144955	0.0009	0.0094	s	31.3	41.8
201693_s_at	*EGR1*	early growth response 1	NM_001964	0.0057	0.0089	s	46.9	65.2
231292_at	*EID3*	EP300 interacting inhibitor of differentiation 3	NM_001008394	0.0002	0.00004	s	38.3	53.5
236700_at	*EIF3C*	eukaryotic translation initiation factor 3, subunit C	NM_001037808	0.0001	0.0007	s	45.1	71.8
1556732_at	*EML4*	Echinoderm microtubule associated protein like 4	NM_001145076	0.0088	0.0060	s	59.6	80.6
1564796_at	*EMP1*	epithelial membrane protein 1	NM_001423	0.0001	0.0028	s	69.8	101.2
209004_s_at	*FBXL5*	F-box and leucine-rich repeat protein 5	NM_012161	0.0008	0.000001	s	29.3	49.8
209189_at	*FOS*	v-fos FBJ murine osteosarcoma viral oncogene homolog	NM_005252	0.0004	0.0020	s	49.6	66.4
220938_s_at	*GMEB1*	glucocorticoid modulatory element binding protein 1	NM_006582	0.0008	0.0024	s	74.7	95.7
233599_at	*hCG_2003663*	hCG2003663	XR_040680	0.0040	0.0092	s	49.7	74.7
215268_at	*KIAA0754*	hypothetical LOC643314	NM_015038	0.0006	0.0068	s	54.9	80.5
227317_at	*LMCD1*	LIM and cysteine-rich domains 1	NM_014583	0.0033	0.0063	s	74.4	108.2
241140_at	*LMO7*	LIM domain 7	NM_005358	0.0001	0.0006	s	39.6	62.6
238771_at	*LOC100129899*	hypothetical protein LOC100129899	XM_001714827	0.0029	0.0099	d	48.4	35.9
213089_at	*LOC100272216*	hypothetical LOC100272216	NR_027439	0.0012	0.0067	s	57.7	87.3
1557987_at	*LOC641298*	SMG1 homolog, phosphatidylinositol 3-kinase-related kinase pseudogene	NR_027154	0.0003	0.0049	s	71.2	95.8
231987_at	*LOC728264*	hypothetical LOC728264	NR_027180	0.0001	0.0060	s	52.7	73.5
201818_at	*LPCAT1*	lysophosphatidylcholine acyltransferase 1	NM_024830	0.0072	0.0003	d	68.0	50.0
215375_x_at	*LRRFIP1*	Leucine rich repeat (in FLII) interacting protein 1	NM_001137550	0.0001	0.0078	s	55.2	70.8
221650_s_at	*MED18*	mediator complex subunit 18	NM_001127350	0.0004	0.0092	s	43.6	60.1
239316_at	*METTL12*	methyltransferase like 12	NM_001043229	0.0003	0.0049	s	64.0	80.2

Affy number	Gene	Protein	RefSeq mRNA	ORIOGEN p-Value	Edge p-Value	Arsenite effect*	Control 4h avg (%)	Arsenite 4h avg (%)
229437_at	*MIRHG2*	microRNA host gene 2 (non-protein coding)	NR_001458	0.0028	0.0039	s	38.0	58.0
217909_s_at	*MLX*	MAX-like protein X	NM_170607	0.0002	0.0016	s	67.4	79.6
243296_at	*NAMPT*	Nicotinamide phosphoribosyltransferase	NM_005746	0.0001	0.0011	s	36.5	50.1
242918_at	*NASP*	Nuclear autoantigenic sperm protein (histone-binding)	NM_002482	0.0001	0.0050	s	61.9	78.2
233870_at	*NAV1*	neuron navigator 1	NM_020443	0.0001	0.0001	s	60.6	81.4
1556277_a_at	*PAPD4*	PAP associated domain containing 4	NM_001114393	0.0014	0.0013	s	71.3	96.8
215418_at	*PARVA*	parvin, alpha	NM_018222	0.0001	0.00001	s	41.7	69.9
1552670_a_at	*PPP1R3B*	protein phosphatase 1, regulatory (inhibitor) subunit 3B	NM_024607	0.0001	0.0009	s	55.8	85.1
204897_at	*PTGER4*	prostaglandin E receptor 4 (subtype EP4)	NM_000958	0.0022	0.0088	s	51.2	61.0
230742_at	*RBM6*	RNA binding motif protein 6	NM_005777	0.0001	0.0008	s	29.4	43.7
228980_at	*RFFL*	ring finger and FYVE-like domain containing 1	NM_001017368	0.0063	0.0033	d	71.7	54.0
204899_s_at	*SAP30*	Sin3A-associated protein, 30kDa	NM_003864	0.0050	0.0014	s	74.4	93.3
215209_at	*SEC24D*	SEC24 family, member D (S. cerevisiae)	NM_014822	0.0001	0.0098	s	30.8	53.4
219677_at	*SPSB1*	splA/ryanodine receptor domain and SOCS box containing 1	NM_025106	0.0042	0.0064	d	74.3	48.1
235925_at	*TCF12*	Transcription factor 12	NM_003205	0.0001	0.0069	s	42.4	59.7
211296_x_at	*UBC*	ubiquitin C	NM_021009	0.0076	0.0002	s	64.5	75.1
227111_at	*ZBTB34*	zinc finger and BTB domain containing 34	NM_001099270	0.0022	0.0022	d	43.3	32.3
217649_at	*ZFAND5*	zinc finger, AN1-type domain 5	NM_001102420	0.0001	0.00003	s	44.8	65.1
234300_s_at	*ZFP28*	zinc finger protein 28 homolog (mouse)	NM_020828	0.0002	0.0046	d	56.2	52.6

*s, stabilized; d, destabilized.
